# Redetermination of *cyclo*-tetra­kis­(μ-5,10,15,20-tetra-4-pyridyl­porphyrinato)tetra­zinc(II) dimethyl­formamide octa­solvate trihydrate at 100 K

**DOI:** 10.1107/S1600536811002054

**Published:** 2011-01-22

**Authors:** Rüdiger W. Seidel, Jürgen Graf, Richard Goddard, Iris M. Oppel

**Affiliations:** a Lehrstuhl für Analytische Chemie, Ruhr-Universität Bochum, Universitätsstrasse 150, 44780 Bochum, Germany; b Incoatec GmbH, Max-Planck-Strasse 2, 21502 Geesthacht, Germany; c Max-Planck-Institut für Kohlenforschung, Kaiser-Wilhelm-Platz 1, 45470 Mülheim an der Ruhr, Germany; d Institut für Anorganische Chemie, Rheinisch-Westfälische Technische Hochschule Aachen, Landoltweg 1, 52074 Aachen, Germany

## Abstract

The structure of the title compound, [Zn_4_(C_40_H_24_N_8_)_4_]·8C_3_H_7_NO·3H_2_O, has been redetermined at 100 K. The redetermination is of significantly higher precision and gives further insight into the disorder of pyridyl groups and solvent mol­ecules. The mol­ecules of (5,10,15,20-tetra-4-pyridyl­porphyrinato)zinc(II) (ZnTPyP) form homomolecular cyclic tetra­mers by coordination of a peripheral pyridyl group to the central Zn atom of an adjacent symmetry-related mol­ecule. The tetra­mer so formed exhibits mol­ecular *S*_4_ symmetry and is located about a crystallographic fourfold rotoinversion axis. Severely disordered dimethyl­formamide and water mol­ecules are present in the crystal, the contributions of which were omitted from refinement. Inter­molecular C—H⋯N hydrogen bonding is observed.

## Related literature

For the structure at 200 K, see: Seidel *et al.* (2010 ▶). For the 2-chloro­phenol solvate of cyclic tetra­meric ZnTPyP, see: Lipstman & Goldberg (2010 ▶). For a review article on structural motifs in coordination polymers of the 5,10,15,20-tetra­4-pyrid­ylporphyrin ligand, see: DeVries & Choe (2009 ▶). For the supra­molecular chemistry of ZnTPyP in the solid-state, see: Lipstman & Goldberg (2010 ▶); Seidel *et al.* (2010 ▶) and references cited therein. For a description of the IμS microfocus X-ray source used in the present study, see: Graf (2008 ▶); Schulz *et al.* (2009 ▶). For *PLATON* / *SQUEEZE*, see: van der Sluis & Spek (1990 ▶); Spek (2009 ▶). For a description of the program *COOT*, see: Emsley *et al.* (2010 ▶).
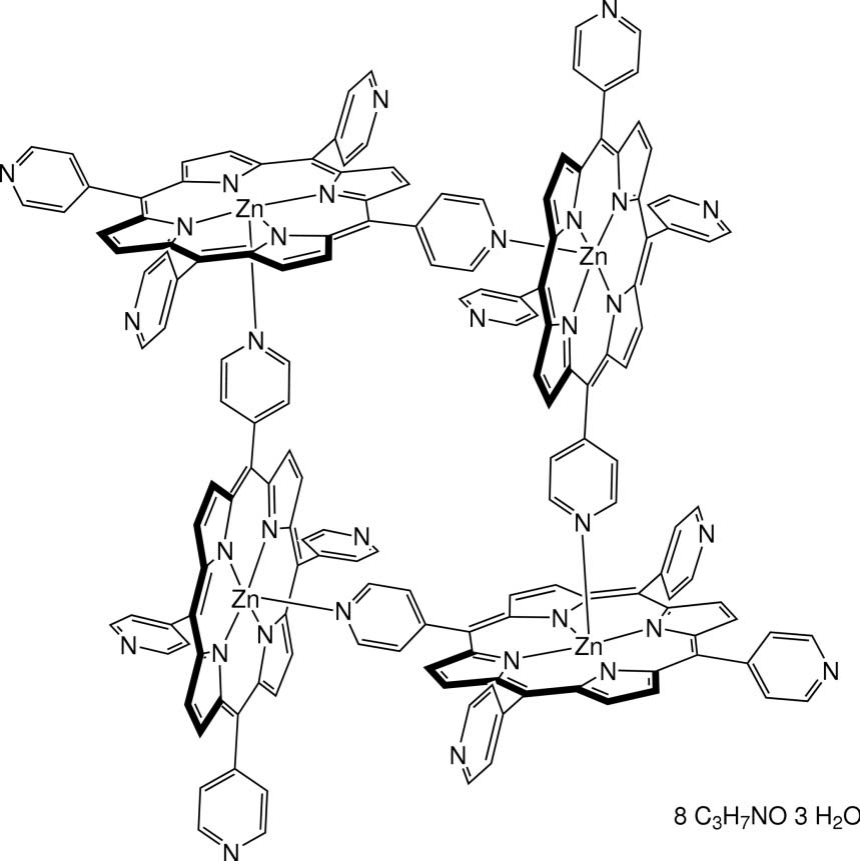


## Experimental

### 

#### Crystal data

[Zn_4_(C_40_H_24_N_8_)_4_]·8C_3_H_7_NO·3H_2_O
*M* *_r_* = 3366.98
Tetragonal, 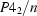

*a* = 23.6897 (5) Å
*c* = 14.9876 (7) Å
*V* = 8411.1 (5) Å^3^
*Z* = 2
Cu *K*α radiation
μ = 1.24 mm^−1^
*T* = 100 K
0.16 × 0.04 × 0.02 mm

#### Data collection

Bruker X8 PROSPECTOR diffractometer
Absorption correction: multi-scan (*SADABS*; Bruker, 2008 ▶) *T*_min_ = 0.827, *T*_max_ = 0.976
44415 measured reflections
7723 independent reflections
6768 reflections with *I* > 2σ(*I*)
*R*_int_ = 0.018

#### Refinement

*R*[*F* ^2^ > 2σ(*F* ^2^)] = 0.042
*wR*(*F* ^2^) = 0.108
*S* = 1.04
7723 reflections
442 parameters
H-atom parameters constrained
Δρ_max_ = 0.59 e Å^−3^
Δρ_min_ = −0.42 e Å^−3^

### 

Data collection: *APEX2* (Bruker, 2008 ▶); cell refinement: *SAINT* (Bruker, 2010 ▶); data reduction: *SAINT*; program(s) used to solve structure: *SHELXS97* (Sheldrick, 2008 ▶); program(s) used to refine structure: *SHELXL97* (Sheldrick, 2008 ▶); molecular graphics: *DIAMOND* (Brandenburg, 2010 ▶); software used to prepare material for publication: *enCIFer* (Allen *et al.*, 2004 ▶).

## Supplementary Material

Crystal structure: contains datablocks global, I. DOI: 10.1107/S1600536811002054/bv2170sup1.cif

Structure factors: contains datablocks I. DOI: 10.1107/S1600536811002054/bv2170Isup2.hkl

Additional supplementary materials:  crystallographic information; 3D view; checkCIF report

## Figures and Tables

**Table:** 

Zn1—N24	2.0684 (15)
Zn1—N21	2.0695 (16)
Zn1—N22	2.0695 (17)
Zn1—N23	2.0747 (16)
Zn1—N101^i^	2.1385 (16)

**Table:** 

N24—Zn1—N21	162.77 (7)
N24—Zn1—N22	88.42 (6)
N21—Zn1—N22	88.84 (7)
N24—Zn1—N23	89.34 (6)
N21—Zn1—N23	87.94 (6)
N22—Zn1—N23	161.70 (7)
N24—Zn1—N101^i^	95.10 (6)
N21—Zn1—N101^i^	102.11 (6)
N22—Zn1—N101^i^	102.00 (6)
N23—Zn1—N101^i^	96.29 (6)

Symmetry code: (i) 
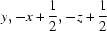
.

**Table 2:** Hydrogen-bond geometry (Å, °)

*D*—H⋯*A*	*D*—H	H⋯*A*	*D*⋯*A*	*D*—H⋯*A*
C7—H7⋯N151^ii^	0.95	2.65	3.583 (4)	167
C17—H17⋯N51^iii^	0.95	2.66	3.583 (3)	165

Symmetry codes: (ii) 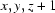; (iii) 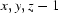.

**Fig. 1.**
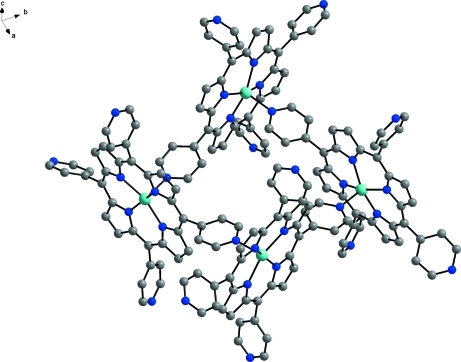
Molecular structure of the title compound. H atoms are omitted for clarity.

**Fig. 2.**
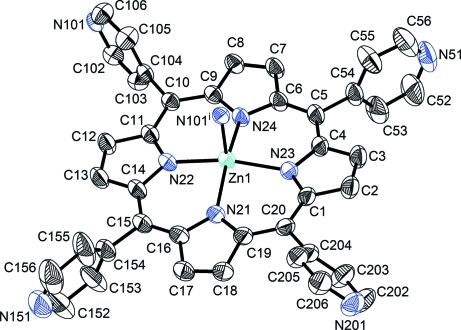
Displacement ellipsoid plot of one repeat unit of cyclic [ZnTPyP]4 drawn at 50% probability. H atoms are omitted for clarity. Symmetry code: (i) y, -x + 1/2, -z + 1/2.

**Fig. 3.**
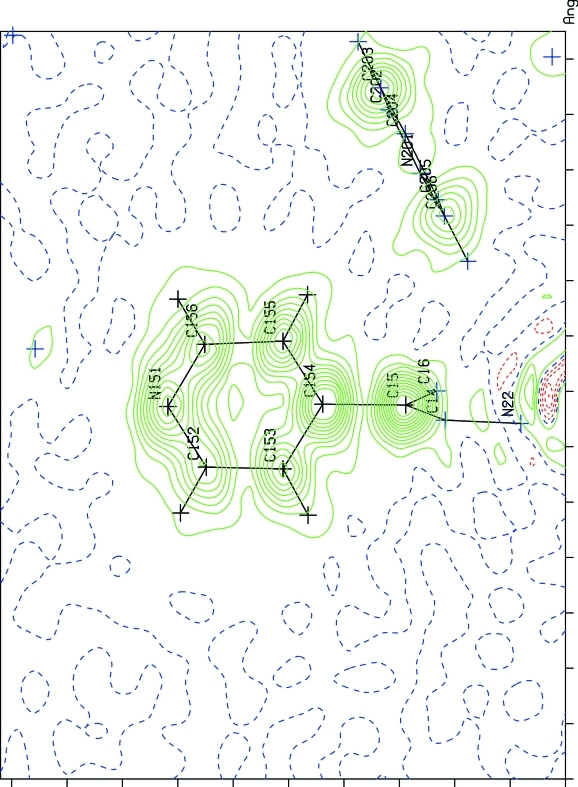
Contour plot of the Fo electron density map in the plane of the pyridyl group attached to C15, calculated with phases from Fc. Contours are drawn at 0.50 e Å-3 starting at 6.00 e Å-3. The contour plot was generated with PLATON (Spek, 2009).

**Fig. 4.**
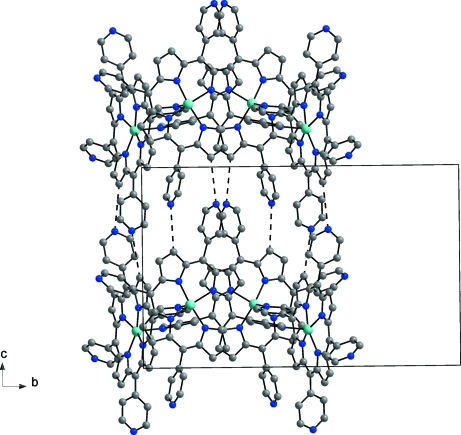
Stacking of the [ZnTPyP]4 entities viewed along the a axis direction. H atoms are omitted for clarity. Cβ—H···Npy interactions are represented by dashed lines.

**Fig. 5.**
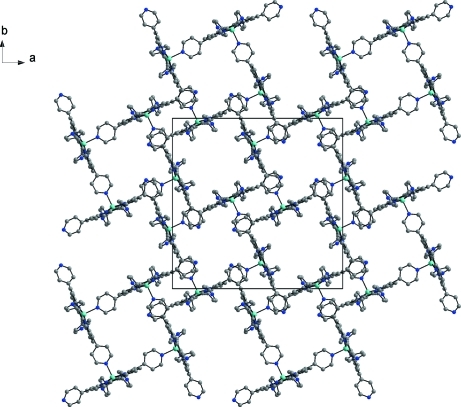
Packing diagram of the title compound projected along the c axis direction. H atoms are omitted for clarity.

**Fig. 6.**
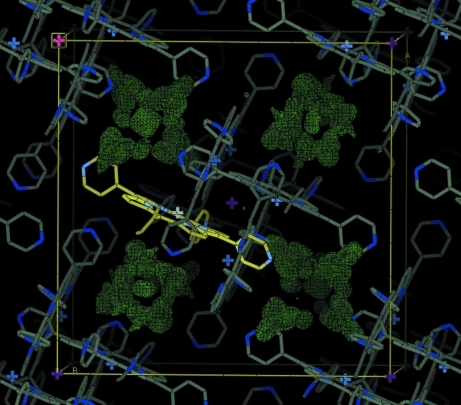
The tetragonal unit cell of the title compound viewed approximately along the c axis direction showing the Fo-Fc map of the disordered solvent regions (contoured at 3.0σ level). The figure was created with COOT (Emsley et al., 2010) using Fo including the contributions of the disordered solvent with phases from Fc based on the model.

## supplementary crystallographic information

### Comment

5,10,15,20-Tetra(4-pyridyl)porphyrin has been widely used as ligand for the construction of coordination polymers (DeVries & Choe, 2009). We and others have reported on the solid-state supramolecular chemistry of the self-complementary [5,10,15,20-tetra(4-pyridyl)porphyrinato]zinc(II) (ZnTPyP) building block (Lipstman & Goldberg, 2010; Seidel *et al.*, 2010 and references cited therein). Recently, we reported the title structure of [ZnTPyP]_4_.

The small dark red plate-shaped crystals of the title compound were subjected to diffraction experiments using a Bruker AXS X8 PROSPECTOR diffractometer equipped with an INCOATEC microfocus X-ray source (IµS) for Cu radiation (Graf, 2008). Such microfocus X-ray sources use multilayer mirrors to focus the X-ray beam onto the crystal and, therefore, lead to a significant reduction of the background and an increase in diffracted intensities. It has already been demonstrated that the Mo IµS gives data of significantly higher quality than a 2 kW Mo fine focus sealed tube, when small crystals are examined (Schulz *et al.*, 2009). The data collection presented here, using the Cu IµS, resulted in intensity data of surprisingly good quality and, hence, indicated a re-refinement of the crystal structure. The crystals investigated in the original work were significantly larger than those examined in the present study and split on cooling to 100 K. For this reason, the data were collected at 200 K with a Cu rotating anode system at that time. Using small crystals has the advantage that these are less likely to split on flash cooling.

The molecular structure of [ZnTPyP]_4_ is depicted in Fig. 1. The asymmetric unit contains one ZnTPyP unit (Fig 2.) and the *S*_4_ symmetric tetramer is generated by crystallographic fourfold rotoinversion symmetry. One peripheral pyridyl group binds to the central Zn atom of an adjacent symmetry related ZnTPyP unit. Zn1 is pentacoordinated and is displaced from the N_4_ mean plane by 0.3196 (9) Å. The coordination geometry parameters about Zn1 are given in Table 1. The three remaining pyridyl groups are non-coordinating. Even at 100 K, the pyridyl groups attached to C5 and C15 show elongated ellipsoids, which cause a checkCIF B level alert (Spek, 2009) due to large *U*_eq_(max)/*U*_eq_(min) ratio. This reveals that the disorder is rather of static than dynamic nature. Attempts were made to describe the electron density of the pyridyl ring attached to C15 (Fig. 3) by a split model. However, the refinement results could not be improved thereby. Thus, both pyridyl rings were finally described with large displacement parameters.

In the crystal, the [ZnTPyP]_4_ entities are stacked into columns located at *x* = 1/4, *y* = 1/4 and *x* = 3/4, *y* = 3/4 (Fig 4). The stacking propagates *via* C_β_—H···N_py_ interactions (see Table 2) by translational symmetry in the *c* axis direction. Within a column, the distance between the centroids of the pyridyl rings attached to C5 and C15^iii^ is 4.0714 (1) Å. Adjacent columns of [ZnTPyP]_4_ are arranged with an offset of *c*/2 (*ca* 7.49 Å). Interstitial channels are formed parallel to the *c* axis direction centred at *x* = 1/4, *y* = 3/4 and *x* = 3/4, *y* = 1/4 (Fig 5). The potential solvent accessible void estimated with *PLATON* / *SOLV* (Spek, 2009) is 33.2% of the unit cell volume. On cooling to 100 K, the *a* lattice vector is shortened by approximately 0.27 Å in comparison to the tetragonal unit cell at 200 K (*a* = 23.958 (2) Å), whereas the length of *c* lattice vector remains relatively unaffected (*c* = 15.0646 (16) Å at 200 K; Seidel *et al.*, 2010).

Despite intensive efforts, the disordered solvent molecules filling the voids within the columns of [ZnTPyP]_4_ and the interstitial channels could not be modeled reasonably with the data collected at 100 K. Nevertheless, residual electron density was visible in a difference Fourier synthesis calculated for the solvent regions (Fig. 6) with phases based on the model using *COOT* (Emsley *et al.*, 2010). For the visualization of the surface of the (difference) electron density using a three-dimensional mesh, the electron densities should be read into *COOT* in terms of structure factors. To obtain a structure factor (.fcf) file containg the informations necessary for the calculation of electron density maps and suitable for *COOT*, the LIST 6 instruction of *SHELXL-97* was used. The atomic model of the framework was read into *COOT* by means of the *SHELXL-97*. res file. The visual inspection of the difference electron density map indicates that four molecules of dimethylformamide (DMF) plus one water molecule are located within the voids in the columns approximately centred at (1/4,1/4,0), whereas another four molecules of DMF and two water molecules are clustered around the 4_2_ screw axes running through the interstitial channels parallel to the *c* axis direction. The compound can, therefore, probably best be described as [ZnTPyP]_4_. 8 DMF. 3 H_2_O. The compound was originally formulated as being a pure DMF solvate (Seidel *et al.*, 2010). To improve the fit of the model to the data and, hence, the precision of the main part of the structure, the contributions of the disordered solvent molecules were removed from the diffraction data with *PLATON* / *SQUEEZE* (van der Sluis & Spek, 1990; Spek, 2009). *SQUEEZE* estimated the electron counts in the voids within the columns and interstitial channels of [ZnTPyP]_4_ to be 182 and 207, respectively. These values are relatively close to those based on the proposed chemical formula (178 and 196).

### Experimental

Small dark red plate-shaped crystals of the title compound were obtained similarly as reported previously (Seidel *et al.*, 2010); 12 mg of ZnTPyP (Aldrich) and 11 mg of [Pd(NO_3_)_2_(en)] (en = 1,2-diaminoethane) were placed in an ampoule and 4 ml of DMF were added. The ampoule was sealed and placed in a heater. The sample was heated to 150 °C in 24 h and held for five days at this temperature. Subsequently, the sample was cooled down to room temperature in 100 h. Noteworthy, the crystals of the title compound were accompanied by crystals of the triclinic phase, containing a polymeric one-dimensional ladder structure of ZnTPyP, as observed previously (Seidel *et al.*, 2010).

### Refinement

For the final refinement, the contributions of severely disordered DMF and water molecules of crystallization were removed from the diffraction data with *PLATON* / *SQUEEZE* (van der Sluis & Spek, 1990; Spek, 2009), see comment. H atoms were placed at geometrically calculated positions and refined with constrained C—H bond length of 0.95 Å and *U*_iso_(H) = 1.2 *U*_eq_(C) allowing them to ride on the parent C atom.

### Crystal data

**Table:**

[Zn_4_(C_40_H_24_N_8_)_4_]·8C_3_H_7_NO·3H_2_O	*D*_x_ = 1.329 Mg m^−^^3^
*M**_r_* = 3366.98	Cu *K*α radiation, λ = 1.54178 Å
Tetragonal, *P*4_2_/*n*	Cell parameters from 130 reflections
Hall symbol: -P 4bc	θ = 3.5–31.5°
*a* = 23.6897 (5) Å	µ = 1.24 mm^−^^1^
*c* = 14.9876 (7) Å	*T* = 100 K
*V* = 8411.1 (5) Å^3^	Plate, dark red
*Z* = 2	0.16 × 0.04 × 0.02 mm
*F*(000) = 3500	

### Data collection

**Table:**

Bruker X8 PROSPECTOR goniometer diffractometer	7723 independent reflections
Radiation source: Incoatec IµS microfocus X-ray source	6768 reflections with *I* > 2σ(*I*)
Incoatec Quazar Multilayer Mirror	*R*_int_ = 0.018
Detector resolution: 8.33 pixels mm^-1^	θ_max_ = 69.2°, θ_min_ = 2.6°
ω scans	*h* = −28→25
Absorption correction: multi-scan (*SADABS*; Bruker, 2008)	*k* = −24→28
*T*_min_ = 0.827, *T*_max_ = 0.976	*l* = −17→14
44415 measured reflections	

### Refinement

**Table:**

Refinement on *F*^2^	Primary atom site location: structure-invariant direct methods
Least-squares matrix: full	Secondary atom site location: difference Fourier map
*R*[*F*^2^ > 2σ(*F*^2^)] = 0.042	Hydrogen site location: inferred from neighbouring sites
*wR*(*F*^2^) = 0.108	H-atom parameters constrained
*S* = 1.04	*w* = 1/[σ^2^(*F*_o_^2^) + (0.0472*P*)^2^ + 5.0454*P*] where *P* = (*F*_o_^2^ + 2*F*_c_^2^)/3
7723 reflections	(Δ/σ)_max_ < 0.001
442 parameters	Δρ_max_ = 0.59 e Å^−^^3^
0 restraints	Δρ_min_ = −0.42 e Å^−^^3^

### Special details

**Table:**

Geometry. All e.s.d.'s (except the e.s.d. in the dihedral angle between two l.s. planes) are estimated using the full covariance matrix. The cell e.s.d.'s are taken into account individually in the estimation of e.s.d.'s in distances, angles and torsion angles; correlations between e.s.d.'s in cell parameters are only used when they are defined by crystal symmetry. An approximate (isotropic) treatment of cell e.s.d.'s is used for estimating e.s.d.'s involving l.s. planes.
Refinement. Refinement of *F*^2^ against ALL reflections. The weighted *R*-factor *wR* and goodness of fit *S* are based on *F*^2^, conventional *R*-factors *R* are based on *F*, with *F* set to zero for negative *F*^2^. The threshold expression of *F*^2^ > σ(*F*^2^) is used only for calculating *R*-factors(gt) *etc*. and is not relevant to the choice of reflections for refinement. *R*-factors based on *F*^2^ are statistically about twice as large as those based on *F*, and *R*- factors based on ALL data will be even larger.

### Fractional atomic coordinates and isotropic or equivalent isotropic displacement parameters (Å^2^)

**Table:**

	*x*	*y*	*z*	*U*_iso_*/*U*_eq_	
Zn1	0.350673 (11)	0.520064 (11)	0.185590 (16)	0.03823 (9)	
N21	0.38601 (8)	0.54998 (7)	0.06850 (10)	0.0433 (4)	
N22	0.27781 (7)	0.50134 (7)	0.11541 (10)	0.0404 (4)	
N23	0.41242 (7)	0.56428 (7)	0.25451 (10)	0.0404 (4)	
N24	0.30440 (7)	0.51392 (7)	0.30222 (10)	0.0377 (4)	
C1	0.46288 (9)	0.58336 (8)	0.22081 (13)	0.0420 (4)	
C2	0.50033 (10)	0.59793 (10)	0.29314 (14)	0.0532 (6)	
H2	0.5379	0.6115	0.2880	0.064*	
C3	0.47175 (10)	0.58861 (10)	0.36945 (15)	0.0534 (6)	
H3	0.4853	0.5949	0.4283	0.064*	
C4	0.41675 (9)	0.56739 (9)	0.34544 (13)	0.0428 (5)	
C5	0.37497 (9)	0.55138 (9)	0.40720 (13)	0.0442 (5)	
C6	0.32280 (9)	0.52641 (9)	0.38657 (12)	0.0411 (4)	
C7	0.28126 (9)	0.50864 (9)	0.45108 (13)	0.0451 (5)	
H7	0.2835	0.5130	0.5140	0.054*	
C8	0.23852 (9)	0.48459 (9)	0.40491 (13)	0.0422 (4)	
H8	0.2052	0.4684	0.4292	0.051*	
C9	0.25323 (8)	0.48825 (8)	0.31165 (12)	0.0362 (4)	
C10	0.21846 (8)	0.46994 (8)	0.24113 (12)	0.0360 (4)	
C11	0.22974 (8)	0.47834 (8)	0.14991 (12)	0.0376 (4)	
C12	0.19052 (9)	0.46543 (9)	0.07858 (13)	0.0460 (5)	
H12	0.1543	0.4486	0.0845	0.055*	
C13	0.21544 (10)	0.48202 (10)	0.00230 (14)	0.0526 (6)	
H13	0.1998	0.4794	−0.0559	0.063*	
C14	0.26979 (9)	0.50435 (10)	0.02480 (13)	0.0477 (5)	
C15	0.30923 (11)	0.52569 (10)	−0.03612 (14)	0.0545 (6)	
C16	0.36281 (10)	0.54737 (10)	−0.01508 (13)	0.0515 (5)	
C17	0.40164 (12)	0.57099 (11)	−0.07905 (15)	0.0636 (7)	
H17	0.3959	0.5743	−0.1416	0.076*	
C18	0.44735 (11)	0.58736 (10)	−0.03328 (15)	0.0581 (6)	
H18	0.4801	0.6046	−0.0575	0.070*	
C19	0.43790 (9)	0.57411 (9)	0.05937 (13)	0.0455 (5)	
C20	0.47569 (9)	0.58754 (8)	0.12914 (13)	0.0429 (5)	
N51	0.40921 (10)	0.58630 (15)	0.68340 (15)	0.0812 (8)	
C52	0.38597 (16)	0.62318 (17)	0.6300 (2)	0.0945 (11)	
H52	0.3762	0.6589	0.6542	0.113*	
C53	0.37466 (15)	0.61389 (13)	0.54115 (18)	0.0816 (9)	
H53	0.3581	0.6429	0.5060	0.098*	
C54	0.38735 (9)	0.56279 (11)	0.50349 (14)	0.0513 (5)	
C55	0.41194 (13)	0.52451 (15)	0.55825 (18)	0.0792 (8)	
H55	0.4222	0.4884	0.5360	0.095*	
C56	0.42214 (14)	0.53814 (18)	0.6469 (2)	0.0885 (10)	
H56	0.4397	0.5105	0.6834	0.106*	
N101	0.06117 (6)	0.38923 (7)	0.30189 (10)	0.0355 (3)	
C102	0.06574 (8)	0.44515 (8)	0.29549 (13)	0.0403 (4)	
H102	0.0327	0.4673	0.3038	0.048*	
C103	0.11567 (8)	0.47254 (8)	0.27747 (13)	0.0406 (4)	
H103	0.1167	0.5126	0.2740	0.049*	
C104	0.16441 (8)	0.44146 (8)	0.26451 (11)	0.0339 (4)	
C105	0.16034 (9)	0.38345 (9)	0.27246 (17)	0.0506 (5)	
H105	0.1928	0.3604	0.2652	0.061*	
C106	0.10845 (9)	0.35925 (9)	0.29112 (16)	0.0490 (5)	
H106	0.1064	0.3194	0.2965	0.059*	
N151	0.2660 (2)	0.5329 (2)	−0.3142 (2)	0.1294 (17)	
C152	0.2458 (3)	0.5685 (2)	−0.2569 (3)	0.155 (2)	
H152	0.2211	0.5972	−0.2780	0.185*	
C153	0.2581 (2)	0.56707 (18)	−0.1666 (2)	0.1290 (18)	
H153	0.2421	0.5945	−0.1278	0.155*	
C154	0.29319 (12)	0.52625 (14)	−0.13291 (16)	0.0729 (8)	
C155	0.31337 (15)	0.4886 (2)	−0.19209 (18)	0.1009 (13)	
H155	0.3375	0.4591	−0.1726	0.121*	
C156	0.29872 (18)	0.4931 (2)	−0.2832 (2)	0.1174 (17)	
H156	0.3133	0.4659	−0.3237	0.141*	
N201	0.63736 (10)	0.65387 (10)	0.05085 (15)	0.0695 (6)	
C202	0.60235 (13)	0.68222 (12)	0.1038 (2)	0.0742 (8)	
H202	0.6140	0.7184	0.1241	0.089*	
C203	0.55016 (12)	0.66270 (10)	0.13114 (18)	0.0635 (7)	
H203	0.5271	0.6852	0.1689	0.076*	
C204	0.53176 (10)	0.61002 (9)	0.10312 (14)	0.0474 (5)	
C205	0.56798 (10)	0.58046 (10)	0.04725 (15)	0.0548 (6)	
H205	0.5577	0.5442	0.0255	0.066*	
C206	0.61911 (11)	0.60416 (12)	0.02354 (17)	0.0645 (7)	
H206	0.6429	0.5831	−0.0153	0.077*	

### Atomic displacement parameters (Å^2^)

**Table:**

	*U*^11^	*U*^22^	*U*^33^	*U*^12^	*U*^13^	*U*^23^
Zn1	0.04778 (16)	0.04374 (16)	0.02318 (14)	−0.00756 (11)	0.00168 (10)	−0.00080 (10)
N21	0.0579 (10)	0.0455 (9)	0.0266 (8)	−0.0116 (8)	0.0027 (7)	0.0007 (7)
N22	0.0485 (9)	0.0476 (9)	0.0251 (8)	−0.0020 (7)	−0.0005 (7)	0.0025 (7)
N23	0.0531 (10)	0.0414 (9)	0.0266 (8)	−0.0084 (7)	0.0028 (7)	−0.0033 (6)
N24	0.0438 (9)	0.0446 (9)	0.0247 (8)	−0.0010 (7)	0.0004 (6)	−0.0035 (6)
C1	0.0521 (12)	0.0395 (10)	0.0342 (10)	−0.0129 (9)	0.0034 (8)	−0.0050 (8)
C2	0.0581 (14)	0.0608 (14)	0.0408 (12)	−0.0204 (11)	0.0039 (10)	−0.0108 (10)
C3	0.0599 (14)	0.0649 (14)	0.0354 (11)	−0.0209 (11)	−0.0001 (10)	−0.0111 (10)
C4	0.0522 (12)	0.0473 (11)	0.0290 (10)	−0.0094 (9)	0.0010 (8)	−0.0072 (8)
C5	0.0520 (12)	0.0531 (12)	0.0275 (10)	−0.0066 (9)	−0.0019 (8)	−0.0055 (8)
C6	0.0479 (11)	0.0494 (11)	0.0259 (10)	0.0000 (9)	0.0024 (8)	−0.0038 (8)
C7	0.0494 (12)	0.0603 (13)	0.0256 (10)	−0.0036 (10)	0.0010 (8)	−0.0026 (9)
C8	0.0458 (11)	0.0528 (12)	0.0279 (10)	0.0012 (9)	0.0035 (8)	−0.0002 (8)
C9	0.0410 (10)	0.0403 (10)	0.0274 (9)	0.0045 (8)	0.0019 (7)	−0.0024 (7)
C10	0.0421 (10)	0.0373 (10)	0.0286 (9)	0.0047 (8)	0.0014 (7)	−0.0020 (7)
C11	0.0428 (10)	0.0414 (10)	0.0287 (10)	0.0024 (8)	−0.0018 (8)	−0.0010 (8)
C12	0.0457 (11)	0.0623 (13)	0.0300 (10)	−0.0037 (10)	−0.0036 (8)	−0.0017 (9)
C13	0.0565 (13)	0.0725 (15)	0.0289 (11)	−0.0093 (11)	−0.0068 (9)	0.0036 (10)
C14	0.0566 (13)	0.0600 (13)	0.0264 (10)	−0.0071 (10)	−0.0044 (9)	0.0036 (9)
C15	0.0687 (15)	0.0670 (15)	0.0279 (11)	−0.0171 (12)	−0.0035 (10)	0.0076 (9)
C16	0.0697 (15)	0.0574 (13)	0.0273 (10)	−0.0158 (11)	0.0024 (9)	0.0052 (9)
C17	0.0816 (18)	0.0817 (17)	0.0276 (11)	−0.0288 (14)	0.0023 (11)	0.0087 (11)
C18	0.0723 (16)	0.0672 (15)	0.0347 (12)	−0.0260 (12)	0.0053 (10)	0.0071 (10)
C19	0.0595 (13)	0.0455 (11)	0.0314 (10)	−0.0118 (9)	0.0050 (9)	0.0012 (8)
C20	0.0570 (12)	0.0366 (10)	0.0351 (10)	−0.0118 (9)	0.0066 (9)	−0.0009 (8)
N51	0.0633 (14)	0.145 (3)	0.0359 (12)	−0.0305 (15)	−0.0006 (10)	−0.0144 (14)
C52	0.126 (3)	0.113 (3)	0.0447 (17)	−0.011 (2)	−0.0056 (17)	−0.0286 (17)
C53	0.126 (3)	0.0787 (19)	0.0403 (14)	−0.0017 (18)	−0.0102 (15)	−0.0181 (13)
C54	0.0499 (12)	0.0755 (16)	0.0286 (11)	−0.0147 (11)	0.0009 (9)	−0.0067 (10)
C55	0.096 (2)	0.100 (2)	0.0419 (15)	0.0139 (17)	−0.0143 (14)	−0.0037 (14)
C56	0.082 (2)	0.135 (3)	0.0486 (17)	0.002 (2)	−0.0152 (14)	0.0081 (18)
N101	0.0379 (8)	0.0442 (9)	0.0244 (8)	0.0040 (7)	−0.0013 (6)	−0.0035 (6)
C102	0.0418 (11)	0.0434 (11)	0.0358 (10)	0.0092 (8)	0.0059 (8)	−0.0024 (8)
C103	0.0466 (11)	0.0392 (10)	0.0360 (10)	0.0058 (8)	0.0063 (8)	0.0009 (8)
C104	0.0386 (10)	0.0412 (10)	0.0218 (8)	0.0052 (8)	−0.0005 (7)	−0.0033 (7)
C105	0.0376 (11)	0.0436 (12)	0.0707 (15)	0.0078 (9)	0.0018 (10)	−0.0042 (10)
C106	0.0422 (11)	0.0384 (11)	0.0665 (15)	0.0038 (9)	−0.0002 (10)	−0.0022 (10)
N151	0.157 (4)	0.182 (4)	0.0499 (18)	−0.092 (3)	−0.022 (2)	0.029 (2)
C152	0.267 (7)	0.130 (4)	0.067 (3)	−0.043 (4)	−0.074 (3)	0.028 (3)
C153	0.218 (5)	0.105 (3)	0.064 (2)	−0.016 (3)	−0.068 (3)	0.026 (2)
C154	0.0797 (18)	0.108 (2)	0.0312 (13)	−0.0441 (16)	−0.0058 (12)	0.0136 (13)
C155	0.088 (2)	0.180 (4)	0.0349 (15)	−0.029 (2)	0.0018 (13)	−0.0193 (18)
C156	0.098 (3)	0.210 (5)	0.0446 (19)	−0.059 (3)	0.0097 (17)	−0.011 (2)
N201	0.0714 (14)	0.0754 (15)	0.0616 (13)	−0.0299 (12)	0.0153 (11)	−0.0022 (11)
C202	0.0847 (19)	0.0592 (15)	0.0786 (19)	−0.0325 (14)	0.0173 (16)	−0.0085 (14)
C203	0.0751 (17)	0.0495 (13)	0.0658 (16)	−0.0190 (12)	0.0165 (13)	−0.0104 (11)
C204	0.0627 (13)	0.0454 (11)	0.0340 (11)	−0.0135 (10)	0.0057 (9)	0.0004 (8)
C205	0.0693 (15)	0.0540 (13)	0.0410 (12)	−0.0153 (11)	0.0148 (10)	−0.0076 (10)
C206	0.0700 (16)	0.0752 (17)	0.0482 (14)	−0.0173 (13)	0.0177 (12)	−0.0064 (12)

### Geometric parameters (Å, °)

**Table:**

Zn1—N24	2.0684 (15)	C19—C20	1.413 (3)
Zn1—N21	2.0695 (16)	C20—C204	1.483 (3)
Zn1—N22	2.0695 (17)	N51—C56	1.302 (5)
Zn1—N23	2.0747 (16)	N51—C52	1.306 (5)
Zn1—N101^i^	2.1385 (16)	C52—C53	1.376 (4)
N21—C19	1.363 (3)	C52—H52	0.9500
N21—C16	1.369 (3)	C53—C54	1.369 (4)
N22—C11	1.364 (3)	C53—H53	0.9500
N22—C14	1.373 (2)	C54—C55	1.355 (4)
N23—C4	1.369 (3)	C55—C56	1.389 (4)
N23—C1	1.374 (3)	C55—H55	0.9500
N24—C9	1.363 (3)	C56—H56	0.9500
N24—C6	1.370 (2)	N101—C102	1.333 (3)
C1—C20	1.411 (3)	N101—C106	1.336 (3)
C1—C2	1.443 (3)	N101—Zn1^ii^	2.1385 (16)
C2—C3	1.347 (3)	C102—C103	1.376 (3)
C2—H2	0.9500	C102—H102	0.9500
C3—C4	1.442 (3)	C103—C104	1.383 (3)
C3—H3	0.9500	C103—H103	0.9500
C4—C5	1.407 (3)	C104—C105	1.383 (3)
C5—C6	1.404 (3)	C105—C106	1.385 (3)
C5—C54	1.497 (3)	C105—H105	0.9500
C6—C7	1.442 (3)	C106—H106	0.9500
C7—C8	1.352 (3)	N151—C152	1.294 (7)
C7—H7	0.9500	N151—C156	1.307 (6)
C8—C9	1.443 (3)	C152—C153	1.385 (5)
C8—H8	0.9500	C152—H152	0.9500
C9—C10	1.408 (3)	C153—C154	1.372 (5)
C10—C11	1.407 (3)	C153—H153	0.9500
C10—C104	1.489 (3)	C154—C155	1.345 (5)
C11—C12	1.449 (3)	C155—C156	1.413 (5)
C12—C13	1.345 (3)	C155—H155	0.9500
C12—H12	0.9500	C156—H156	0.9500
C13—C14	1.432 (3)	N201—C206	1.320 (3)
C13—H13	0.9500	N201—C202	1.330 (4)
C14—C15	1.401 (3)	C202—C203	1.382 (4)
C15—C16	1.405 (3)	C202—H202	0.9500
C15—C154	1.500 (3)	C203—C204	1.387 (3)
C16—C17	1.442 (3)	C203—H203	0.9500
C17—C18	1.339 (3)	C204—C205	1.388 (3)
C17—H17	0.9500	C205—C206	1.382 (3)
C18—C19	1.441 (3)	C205—H205	0.9500
C18—H18	0.9500	C206—H206	0.9500
			
N24—Zn1—N21	162.77 (7)	C17—C18—H18	126.1
N24—Zn1—N22	88.42 (6)	C19—C18—H18	126.1
N21—Zn1—N22	88.84 (7)	N21—C19—C20	126.29 (18)
N24—Zn1—N23	89.34 (6)	N21—C19—C18	109.15 (18)
N21—Zn1—N23	87.94 (6)	C20—C19—C18	124.46 (19)
N22—Zn1—N23	161.70 (7)	C1—C20—C19	124.66 (19)
N24—Zn1—N101^i^	95.10 (6)	C1—C20—C204	118.30 (18)
N21—Zn1—N101^i^	102.11 (6)	C19—C20—C204	116.97 (18)
N22—Zn1—N101^i^	102.00 (6)	C56—N51—C52	115.3 (3)
N23—Zn1—N101^i^	96.29 (6)	N51—C52—C53	124.6 (3)
C19—N21—C16	106.82 (16)	N51—C52—H52	117.7
C19—N21—Zn1	126.42 (13)	C53—C52—H52	117.7
C16—N21—Zn1	126.72 (14)	C54—C53—C52	119.8 (3)
C11—N22—C14	106.27 (17)	C54—C53—H53	120.1
C11—N22—Zn1	126.10 (13)	C52—C53—H53	120.1
C14—N22—Zn1	127.37 (14)	C55—C54—C53	115.9 (2)
C4—N23—C1	106.45 (16)	C55—C54—C5	123.2 (2)
C4—N23—Zn1	125.15 (13)	C53—C54—C5	120.9 (2)
C1—N23—Zn1	126.61 (13)	C54—C55—C56	119.9 (3)
C9—N24—C6	106.48 (15)	C54—C55—H55	120.0
C9—N24—Zn1	126.17 (12)	C56—C55—H55	120.0
C6—N24—Zn1	126.59 (13)	N51—C56—C55	124.4 (3)
N23—C1—C20	124.64 (18)	N51—C56—H56	117.8
N23—C1—C2	109.72 (17)	C55—C56—H56	117.8
C20—C1—C2	125.64 (19)	C102—N101—C106	116.85 (17)
C3—C2—C1	106.8 (2)	C102—N101—Zn1^ii^	120.26 (13)
C3—C2—H2	126.6	C106—N101—Zn1^ii^	122.55 (14)
C1—C2—H2	126.6	N101—C102—C103	123.56 (18)
C2—C3—C4	107.42 (19)	N101—C102—H102	118.2
C2—C3—H3	126.3	C103—C102—H102	118.2
C4—C3—H3	126.3	C102—C103—C104	119.61 (18)
N23—C4—C5	126.00 (18)	C102—C103—H103	120.2
N23—C4—C3	109.56 (18)	C104—C103—H103	120.2
C5—C4—C3	124.42 (18)	C105—C104—C103	117.31 (18)
C6—C5—C4	125.98 (18)	C105—C104—C10	122.03 (17)
C6—C5—C54	117.43 (18)	C103—C104—C10	120.65 (17)
C4—C5—C54	116.59 (18)	C104—C105—C106	119.38 (19)
N24—C6—C5	125.05 (18)	C104—C105—H105	120.3
N24—C6—C7	109.78 (17)	C106—C105—H105	120.3
C5—C6—C7	125.15 (18)	N101—C106—C105	123.3 (2)
C8—C7—C6	106.91 (17)	N101—C106—H106	118.4
C8—C7—H7	126.5	C105—C106—H106	118.4
C6—C7—H7	126.5	C152—N151—C156	117.0 (4)
C7—C8—C9	106.83 (18)	N151—C152—C153	123.7 (5)
C7—C8—H8	126.6	N151—C152—H152	118.2
C9—C8—H8	126.6	C153—C152—H152	118.2
N24—C9—C10	125.42 (17)	C154—C153—C152	120.3 (5)
N24—C9—C8	110.00 (16)	C154—C153—H153	119.9
C10—C9—C8	124.54 (18)	C152—C153—H153	119.9
C11—C10—C9	125.06 (18)	C155—C154—C153	116.1 (3)
C11—C10—C104	117.12 (16)	C155—C154—C15	122.8 (3)
C9—C10—C104	117.77 (16)	C153—C154—C15	121.1 (3)
N22—C11—C10	125.68 (17)	C154—C155—C156	120.0 (4)
N22—C11—C12	109.87 (17)	C154—C155—H155	120.0
C10—C11—C12	124.42 (18)	C156—C155—H155	120.0
C13—C12—C11	106.50 (19)	N151—C156—C155	122.9 (5)
C13—C12—H12	126.7	N151—C156—H156	118.6
C11—C12—H12	126.7	C155—C156—H156	118.6
C12—C13—C14	107.59 (19)	C206—N201—C202	115.6 (2)
C12—C13—H13	126.2	N201—C202—C203	124.5 (2)
C14—C13—H13	126.2	N201—C202—H202	117.8
N22—C14—C15	124.8 (2)	C203—C202—H202	117.8
N22—C14—C13	109.76 (18)	C202—C203—C204	119.5 (2)
C15—C14—C13	125.41 (19)	C202—C203—H203	120.3
C14—C15—C16	126.03 (19)	C204—C203—H203	120.3
C14—C15—C154	117.7 (2)	C203—C204—C205	116.2 (2)
C16—C15—C154	116.28 (19)	C203—C204—C20	121.7 (2)
N21—C16—C15	125.76 (19)	C205—C204—C20	122.07 (19)
N21—C16—C17	109.5 (2)	C206—C205—C204	119.5 (2)
C15—C16—C17	124.7 (2)	C206—C205—H205	120.3
C18—C17—C16	106.7 (2)	C204—C205—H205	120.3
C18—C17—H17	126.6	N201—C206—C205	124.8 (2)
C16—C17—H17	126.6	N201—C206—H206	117.6
C17—C18—C19	107.8 (2)	C205—C206—H206	117.6

Symmetry codes: (i) *y*, −*x*+1/2, −*z*+1/2; (ii) −*y*+1/2, *x*, −*z*+1/2.

### Hydrogen-bond geometry (Å, °)

**Table:**

*D*—H···*A*	*D*—H	H···*A*	*D*···*A*	*D*—H···*A*
C7—H7···N151^iii^	0.95	2.65	3.583 (4)	167.
C17—H17···N51^iv^	0.95	2.66	3.583 (3)	165.

Symmetry codes: (iii) *x*, *y*, *z*+1; (iv) *x*, *y*, *z*−1.
